# Genomics of host-pathogen interactions: challenges and opportunities across ecological and spatiotemporal scales

**DOI:** 10.7717/peerj.8013

**Published:** 2019-11-05

**Authors:** Kathrin Näpflin, Emily A. O’Connor, Lutz Becks, Staffan Bensch, Vincenzo A. Ellis, Nina Hafer-Hahmann, Karin C. Harding, Sara K. Lindén, Morten T. Olsen, Jacob Roved, Timothy B. Sackton, Allison J. Shultz, Vignesh Venkatakrishnan, Elin Videvall, Helena Westerdahl, Jamie C. Winternitz, Scott V. Edwards

**Affiliations:** 1 Department of Organismic and Evolutionary Biology and Museum of Comparative Zoology, Harvard University, Cambridge, MA, United States of America; 2 Molecular Ecology and Evolution Lab, Department of Biology, Lund University, Lund, Sweden; 3 Aquatic Ecology and Evolution, Limnological Institute University Konstanz, Konstanz, Germany; 4 Department of Evolutionary Ecology, Max Planck Institute for Evolutionary Biology, Plön, Germany; 5 EAWAG, Swiss Federal Institute of Aquatic Science and Technology, Dübendorf, Switzerland; 6 Department of Biological and Environmental Sciences, University of Gothenburg, Gothenburg, Sweden; 7 Gothenburg Centre for Advanced Studies in Science and Technology, Chalmers University of Technology and University of Gothenburg, Gothenburg, Sweden; 8 Department of Medical Chemistry and Cell Biology, Institute of Biomedicine, Sahlgrenska Academy, University of Gothenburg, Gothenburg, Sweden; 9 Section for Evolutionary Genomics, Natural History Museum of Denmark, Department of Biology, University of Copenhagen, Copenhagen, Denmark; 10 Informatics Group, Harvard University, Cambridge, MA, United States of America; 11 Ornithology Department, Natural History Museum of Los Angeles County, Los Angeles, CA, United States of America; 12 Center for Conservation Genomics, Smithsonian Conservation Biology Institute, National Zoological Park, Washington, DC, United States of America; 13 Department of Animal Behaviour, Bielefeld University, Bielefeld, Germany

**Keywords:** *Plasmodium*, MHC, Immunotoxins, Mucus, Natural selection, GWAS, Infectious diseases, Anthropogenic stressors, Co-evolution, Epidemiological surveillance

## Abstract

Evolutionary genomics has recently entered a new era in the study of host-pathogen interactions. A variety of novel genomic techniques has transformed the identification, detection and classification of both hosts and pathogens, allowing a greater resolution that helps decipher their underlying dynamics and provides novel insights into their environmental context. Nevertheless, many challenges to a general understanding of host-pathogen interactions remain, in particular in the synthesis and integration of concepts and findings across a variety of systems and different spatiotemporal and ecological scales. In this perspective we aim to highlight some of the commonalities and complexities across diverse studies of host-pathogen interactions, with a focus on ecological, spatiotemporal variation, and the choice of genomic methods used. We performed a quantitative review of recent literature to investigate links, patterns and potential tradeoffs between the complexity of genomic, ecological and spatiotemporal scales undertaken in individual host-pathogen studies. We found that the majority of studies used whole genome resolution to address their research objectives across a broad range of ecological scales, especially when focusing on the pathogen side of the interaction. Nevertheless, genomic studies conducted in a complex spatiotemporal context are currently rare in the literature. Because processes of host-pathogen interactions can be understood at multiple scales, from molecular-, cellular-, and physiological-scales to the levels of populations and ecosystems, we conclude that a major obstacle for synthesis across diverse host-pathogen systems is that data are collected on widely diverging scales with different degrees of resolution. This disparity not only hampers effective infrastructural organization of the data but also data granularity and accessibility. Comprehensive metadata deposited in association with genomic data in easily accessible databases will allow greater inference across systems in the future, especially when combined with open data standards and practices. The standardization and comparability of such data will facilitate early detection of emerging infectious diseases as well as studies of the impact of anthropogenic stressors, such as climate change, on disease dynamics in humans and wildlife.

## Introduction

Pathogens are widely agreed to be among the strongest agents of natural selection in nature, and their influence on the genomes of host species is often readily detectable (Kosiol et al., 2008; Enard et al., 2016; Quach et al., 2016; Shultz & Sackton, 2019). With the advent of rapid DNA sequencing technologies, genetic studies of host-pathogen interactions have moved from single gene perspectives to genome-wide approaches interrogating whole genomes of hosts and/or pathogens. At the same time, these studies have begun to tackle an increasingly diverse array of systems in both the field and laboratory, and have expanded from analysis of single pathogens to multiple pathogens under a variety of conditions. Environmental factors and gene-by-environment interactions, such as those beginning to be studied in microbiome research (Libertucci & Young, 2019), are increasingly appreciated as important in modulating the severity and fitness consequences of infections (Sekirov et al., 2010; Kamada et al., 2013; Villarino et al., 2016; Chomwong et al., 2018). As genomic approaches become increasingly accessible and affordable, it is becoming clear that the limiting factor in host-pathogen research is often not the technical aspects of sequencing pathogens or host genomes, but rather the ecological, immunological and epigenetic context in which genomic data are embedded (Kratochwil & Meyer, 2015). To mention one example, post-translational modifications of proteins in the mucus are known to play critical roles in pathogen defense in addition to genetic factors (Lindén et al., 2008b; Linden et al., 2008a).

**Figure 1:**
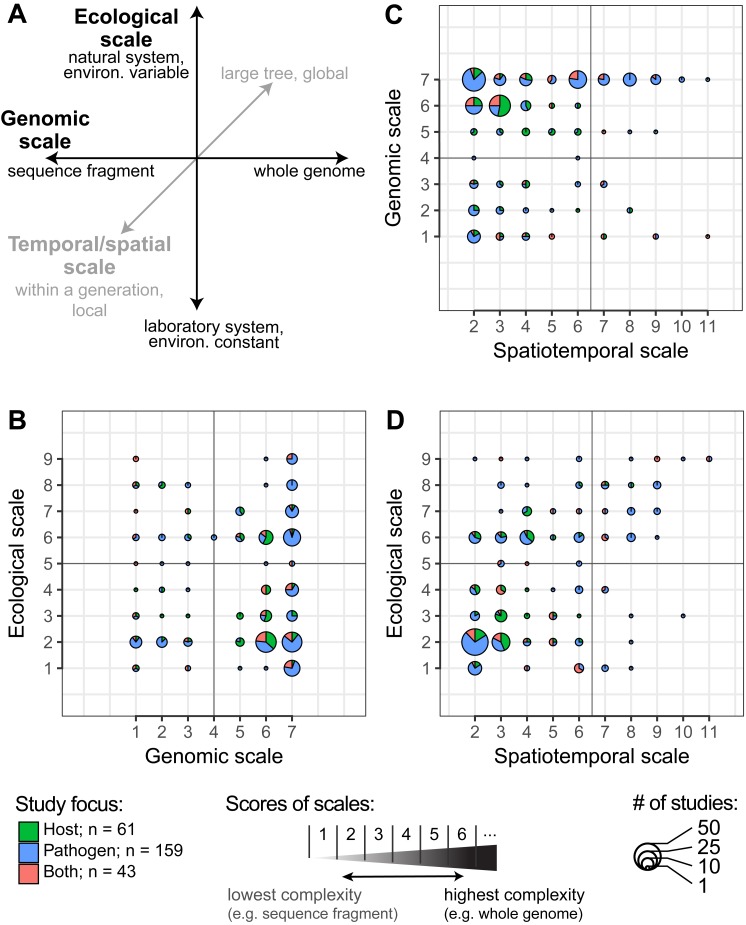
The diversity of recent studies of host-pathogen interactions. (A) Each of three scales of complexity—genomic, ecological and spatiotemporal—is represented as an axis in this illustration. A study of host-pathogen interaction is placed into this three-dimensional space based on the level of genetic, ecological, and spatiotemporal detail that is being studied (see Table 1 for scores of scales). (B–D) Pie charts summarize the results of the scores for the level of genetic, ecological, and spatiotemporal complexity investigated in host-pathogen studies published between 2014–2018. (B) The complexity of the ecological and genomic settings across studies are not correlated (Spearman’s *ρ* = 0.02, *p*-value adjusted = 1.00; (C) nor are the genomic and spatiotemporal scale (*ρ* = 0.16, *p*-value adj. = 0.13. (D) In contrast, the ecological scale positively correlates with the score of spatiotemporal scale across studies (*ρ* = 0.50, *p*-value adj. = 0.00).

Host-pathogen studies encompass an extraordinary variety of temporal and spatial scales, including wide ranges of ecological settings and pathogen complexities—such as experimental versus field studies or single versus multiple pathogens—as well as genomic complexities, ranging from candidate gene studies to whole genome scans (Fig. 1A). Any single study can be classified according to these scales, with concomitant benefits and deficiencies in capturing the details of host-pathogen interactions in the real world. For example, studies aiming to link the evolution of host and pathogen genomes and to detect genomic signatures of host-pathogen interactions have arisen from searches for associations with single host candidate genes, such as genes of the major histocompatibility complex (MHC) (Hill et al., 1991; Kaslow et al., 1996; Wegner, Reusch & Kalbe, 2003; Meyer-Lucht & Sommer, 2005; Savage & Zamudio, 2011), to genome-wide scans for associations with resistance or susceptibility (e.g., Fumagalli et al., 2011; Bartha et al., 2013). We now have genomic insights into host-pathogen interactions that stem from field studies investigating temporal and spatial patterns (Hill et al., 1991; Savage & Zamudio, 2011; Penczykowski, Laine & Koskella, 2015; Bourgeois et al., 2017); to experimentally evolving populations or ancient DNA studies encompassing hundreds or thousands of host generations (Bos et al., 2011; Cairns et al., 2017; Tso et al., 2018; Spyrou et al., 2019); to phylogenetic and comparative studies spanning tens of millions of years (Enard et al., 2016; Koonin, Makarova & Wolf, 2017; Shultz & Sackton, 2019). This variety makes it challenging to draw broad generalizations linking processes on different scales and, to date, few syntheses have attempted to bridge the many temporal and spatial scales on which host-pathogen studies take place. This represents an important deficit, as generalizability is key to identifying fundamental principles in the field of host-pathogen interactions and enables accurate predictions to be made about host-pathogen interactions in new study systems.

In this perspective, we aim to address the complexities and commonalities of diverse studies of host-pathogen interactions through the lens of evolutionary genomics. We emphasize the wide range of approaches used recently and focus primarily on evolutionary responses of hosts to pathogens (Fig. 1). We first document the diversity of recent studies of host-pathogen interactions through a comprehensive analysis of the recent literature on the subject. This survey documents the sheer diversity of temporal and spatial scales on which host-pathogen studies are conducted, but also reveals that the heterogeneity of results across studies, from laboratory to field to experimental settings, poses a challenge for synthesis. Our survey identifies gaps in emphasis on research on host-pathogen interactions, but also reveals opportunities for discovering common principles and methodologies that are likely to drive the research field forward. We then review major themes in the study of interactions between hosts and pathogens in the wild. While daunting in terms of confounding variables, such studies provide opportunities for studying the synergistic effects of anthropogenic change and the evolutionary response to epizootics. At the same time, an increasing number of experimental studies that examine the effects of multiple interacting pathogens on their hosts, or of host microbiome on infection outcome, capture some of the reality of epizootics in nature. We conclude that the full promise of genomic and other -omics approaches to further our understanding of host-pathogen interactions will not be realized until these data are thoroughly and consistently embedded in high quality, consistent, and reproducible ecological and environmental metadata. Increased resolution of ecological metadata, smart databases that facilitate collaboration and comparisons across studies, and deposition of voucher specimens associated with specific studies in museum collections are just some of the ways in which genomic data can realize their full potential. These new tools will facilitate the application of knowledge of basic principles of host-pathogen interactions to real world problems affecting wildlife, endangered species, and ultimately human welfare.

### Survey methodology

This perspective piece is the result of discussions held during the “Origins of Biodiversity Workshop” organized during May 29–June 2, 2017 by Chalmers University of Technology and the University of Gothenburg, Sweden, under the auspices of the Gothenburg Centre for Advanced Studies (GoCAS). We gathered international scholars and students with interdisciplinary backgrounds to discuss future perspectives of the study of host-pathogen co-evolution in the genomic era. During the workshop we identified major directions that have been enabled by advances in genomic techniques and in particular we highlight the resulting diversity of host-pathogen studies in their ecological, temporal and genomic detail at which they are studied. Our goal is not to provide a complete overview of the host-pathogen literature, but rather to illustrate the diversity of recent research undertaken in the field and the associated challenges towards a comparable and inter-communicative understanding of causes and consequences of host-pathogen interactions across systems. To quantify currently studied dimensions (ecological complexity, spatiotemporal scope and genomic scale; Fig. 1A) of host-pathogen research we conducted a literature search on Web of Science (accessed August 30, 2018) with the following search query: (host-parasite* OR host-pathogen*) AND (genomic*). We refined the search hits by document type to include only articles, covering the publication years 2014–2018. Of a total of 341 screened publications from this search, 10 publications were excluded due to access problems, 12 publications were unrelated to host-pathogen research, 48 publications were identified as review articles, and an additional 8 publications were excluded because no genomic aspect was present. In total, we scored and evaluated 263 papers based on broadly defined categories for each scale defined in Table 1. The categories were chosen to represent a rough continuous scale along the genomic, ecological, and spatiotemporal axes (Fig. 1A). We used Spearman’s rho to assess the rank based association between scales and adjusted *p*-values for multiple testing (Benjamini & Hochberg, 1995). The reference list and scoring results are listed in Table S1.

**Table 1:** Definition of categories for each scale and assigned scores used for the evaluation of host-pathogen literature.

Score^a^	**Genomic scale**	**Ecological scale**	**Temporal scale^b^**	**Spatial scale^b^**
1	gene/ sequence fragment	none/ theoretical	none	none
2	full gene/ regulator	single species, laboratory system, environment constant	single generation	local (one population)
3	gene family/ microsatellite	single species, laboratory system, environment variable	few generations	intermediate (couple of populations)
4	whole plastid genome	multiple species, laboratory system, environment constant	many generations	species range
5	reduced genome representation	multiple species, laboratory system, environment variable	speciation time (small tree)	global
6	exome/ transcriptome/ proteome	single species, natural system, environment constant	speciation time (large tree)	
7	whole genome	single species, natural system, environment variable		
8		multiple species, natural system, environment constant		
9		multiple species, natural system, environment variable		

**Notes.**

a see Table S1 for list of references and associated scoring results.

b the spatiotemporal scale (Fig. 1) is the sum of the individual scores of the temporal and spatial scales.

### Understanding the diversity of host-pathogen studies across genomic, ecological and spatiotemporal scales

We have outlined in the introduction that the published literature on host-pathogen interaction spans a diverse range of genomic, ecological and spatiotemporal scales. However, how the current published literature is distributed within this multidimensional space has not, until now, been mapped out (Fig. 1A). To understand the range of investigation at the genomic, ecological and spatiotemporal scale in recent studies on host-pathogen systems, and to discern where gaps in recent efforts might persist, we performed a literature search to classify and quantify the distribution of studies across these three scales. For this, we reviewed 263 studies of host-pathogen interactions published in the period between 2014–2018 (see Table 1 for scoring categories and Survey Methodology for specific details). A better understanding of the current placement of host-pathogen studies should help us gain a better insight into how genomics has contributed and will continue to contribute to the understanding of host-pathogen interactions from the perspectives of hosts or pathogens and at various levels of biological detail.

We scored each study on three scales: genomic complexity, temporal and spatial complexity, and ecological complexity (see Table 1 for the scoring key and definition). We found that high genomic resolution (mean score = 5.4 ± 2.1 SD, range = 1–7) at the level of the whole genome is employed to investigate questions that span the whole range of ecological scales, from theory, laboratory systems, and to multi-species natural systems with environmental variability (mean score = 4.1 ± 2.4 SD, range = 1–9; Fig. 1B). Investigations of pathogen genomics dominate the dataset, whereas whole genome investigations of hosts are less common and more often examine a reduced representation of the genome (e.g., ddRADSeq, sequence capture, genotype by sequencing), transcriptome, or proteome. Studies encompassing the interaction of both host and pathogens simultaneously are rare. Genomic techniques are rarely used to address complex spatiotemporal scales (mean score = 4.1 ± 2.3 SD, range = 2–11), such as throughout the geographical range of a species, or across multiple different species. (Fig. 1C). Intriguingly, with increasing ecological complexity in a study, more complex spatiotemporal scales are also addressed (Fig. 1D). However, only a few studies are classified as complex in terms of spatiotemporal setting: across all studies spatial (mean score = 1.6 ± 1.0 SD, range = 1–5) and temporal scores (mean score = 2.4 ± 1.7 SD, range = 1–6) are on average low. In particular, studies of complex spatial scales, such as interrogation across multiple populations across a species’ range, are virtually missing. An overview of the general advantages and disadvantages of different genomic, ecological, temporal and spatial scales are summarized in Table 2.

**Table 2:** Overview of the advantages and disadvantages of studies conducted at different genomic, ecological, temporal and spatial scales.

Category	Scale	Advantages	Disadvantages
Genomic scale	Narrow e.g., single gene	Known function	Limited information
	Broad e.g., whole genome	Discover significant genomic regions	Interpretation limited by annotation
Ecological scale	Narrow e.g., single species	Feasibility of detailed study	Information may be restricted to study system
	Broad e.g., multiple species	Generalizability; more ‘realistic’ insights	Limitation on depth of study
Temporal scale	Narrow e.g., within single generation	Feasibility of detailed study	Temporal patterns not detected or restricted to ecological time scales
	Broad e.g., across species (evolutionary time)	Ability to detect macroevolutionary patterns	Detail of within-species processes may be lacking; feasibility
Spatial scale	Narrow e.g., single population	Feasibility of detailed study	Limited ability to generalize across broader spatial contexts
	Broad e.g., global	Identify general patterns	Feasibility

The evaluation of published studies on host-pathogen systems not only reveals the expected recent increase in whole genome datasets for a broad range of host-pathogen studies, but also what is missing when addressing complex systems on ecological and spatiotemporal scales. This highlights a gap that future efforts might be specifically focused on in order to be able to project and test whether the identified underlying genetics of host-pathogen interactions scale-up from simple systems, such as a laboratory study, to ecological and spatiotemporal complex situations in the wild. The quantity and complexity of the sequence data generated in many recent and ongoing studies of host-pathogen interactions presents a unique opportunity for further investigations addressing as yet unexplored aspects of the data. Perhaps most critically, however, we suspect that this massive volume of complex data poses an increasing challenge for comparisons across studies. The lack of comprehensive cross-taxon comparative databases of host-pathogen interactions likely impedes the synthesis of individual host-pathogen studies and translation of new knowledge into solutions for real world problems. In the real-world, (a) pathogens attack hosts in the context of changing host environments, (b) these environments are increasingly impacted by anthropogenic forces such as climate change, and (c) are usually characterized by diverse communities of pathogenic and non-pathogenic organisms. Our cross-section of recent studies of host-pathogen interactions suggests that these complexities are rarely captured in a single study. Thus, it will be essential to conduct comparative studies and perform meta-analyses of existing data across systems in order to achieve a comprehensive synthesis of how genomics can address host-pathogen interactions at different scales. In the following sections, we aim to highlight a few examples of challenges and opportunities and conclude with a suggestion of how integration across studies might be more successfully achieved through improved data and workflow documentation.

### Disentangling hidden histories in genes and genomes of hosts and pathogens

**Figure 2:**
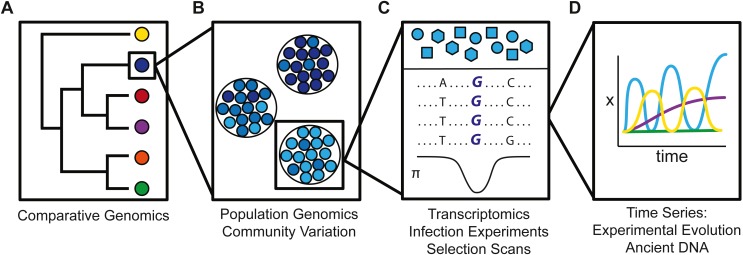
Schematic illustration how genetic variation varies (A) across species, (B) across populations, (C) within a population, and (D) on an ecological time scale. (A) Comparative genomics across species can be used to identify genomic loci consistently under positive selection in particular lineages or all lineages. (B) Across populations, population genomic variation in different geographic populations can be correlated with pathogen communities. (C) Within a single population, phenotypic variation among individuals can be linked to pathogen variation or differentially expressed genes with transcriptomics. Genome scans may also identify regions of the genome under selection. (D) Finally, time series either derived through experimental evolution or studies of ancient DNA or diachronic samples can be used to identify the dynamics of a phenotype or allele frequency through time.

The pathogenic lifestyle is ubiquitous across the tree of life, and pathogens are estimated to represent a substantial proportion of the diversity and biomass of many ecosystems (Windsor, 1998; Dobson et al., 2008; Kuris et al., 2008; Poulin, 2014; Padra et al., 2018). PCR-based technologies and the advent of high throughput sequencing, along with the associated reduction in sequencing costs, have facilitated the description of novel pathogens (Woo et al., 2008; Lipkin, 2013; Bullman, Meyerson & Kostic, 2017; Titcomb, Jerde & Young, 2019), with particular success in viral pathogen discovery (Chiu, 2013; Datta et al., 2015). Furthermore, the application of metagenomic approaches has highlighted complex host-pathogen interactions and implicated host- and pathogen-associated microbial communities in successful pathogen infection and disease development (Sekirov et al., 2010; Kamada et al., 2013). This suggests that a pathogen rarely occurs alone, and instead may commonly be a member of a larger community (Robinson, Bohannan & Young, 2010; Schmid-Hempel, 2011; Gregory et al., 2019). Hence, understanding the interplay between multiple pathogens and associated microbiomes requires disentangling several levels of complexity. It is also crucial to gain an understanding of the fitness effects of each putative pathogen on its host, because the magnitude of the fitness cost (i.e., virulence) of a pathogen during infection determines its place on the mutualist-pathogen continuum. In principal, this requires demonstration of a fitness cost to the host, yet demonstrating fitness effects of many putative pathogens in nature is challenging and often requires datasets that are much larger than those obtained in a typical field study (see Box 1). Importantly, the fact that measuring the fitness consequences of infections in wild animals is challenging does not imply that pathogens are insignificant selective agents in the evolution of host genomes. In fact, pathogens are widely presumed to be among the strongest selective agents (Fumagalli et al., 2011; Pittman et al., 2016). Mutations conferring moderate or large benefits of resistance to hosts can become readily fixed by selection and are detectable through genome scans (Nielsen, 2005; Vitti, Grossman & Sabeti, 2013). Thus, comparative and population genetic studies of host genomes present compelling approaches for studying the presumed impact of pathogens (Fig. 2).

BOX 1 Demonstrating pathogen-induced fitness costs in the wild.

Determining where an organism lies on the mutualist-pathogen continuum requires an assessment of the fitness costs (i.e., virulence) elicited by a putative pathogen when it has established itself within its host in its natural habitat. In such a scenario, the feasibility of estimating fitness costs strongly depends, on the one hand, on the magnitude of the fitness effect and, on the other hand, the sample size. For example, severe negative fitness effects in birds due to infections by the introduced malaria pathogen *Plasmodium relictum*, have been readily demonstrated in several species of Hawaiian honeycreepers (*Drepanididae*) (Van Riper et al., 1986)). However, when *P. relictum* infects host species with which it has presumably co-evolved, observed fitness costs are lower (Bensch et al., 2007). Thus, hypothetically, demonstrating a negative fitness effect (i.e., mortality) of 5% year-on-year in natural populations (assuming a pathogen prevalence of 20% and an annual background mortality of 50%) requires a sample size of more than 2,000 host individuals and the ability to accurately measure individual survival. The situation becomes even more complex when hosts are repeatedly exposed to the same pathogen and mortality varies across exposures. For example, if mortality is highest upon primary infection, in year two individuals that were unexposed in year one will be at a higher risk of dying than individuals that have been previously exposed. Often only long-term studies, such as that conducted by Asghar et al. (2015) on the effects of *Plasmodium* on lifetime fitness and survival of Great Reed Warblers, provide the sensitivity required to detect fitness costs.

Given that the ecological role of an organism can be dynamic, the fitness consequences for a host of a particular pathogen are strongly dependent on the environmental and genetic context. The most obvious illustration of this is variation in virulence associated with host switches: *Mycoplasma* infection in house finches as compared to other song birds (Ley et al., 2016), Ebola virus in humans as compared to bats (Leroy et al., 2005), or the morbillivirus Phocine Distemper Virus (PDV) in harbor seals as compared to other Arctic pinniped species (Härkönen et al., 2006) are all cases where virulence dramatically increased after switching to a new host. Second, another level of complexity presents itself in the cases of complex pathogen life cycles, where pathogens may require multiple host species for different developmental stages in order to complete their life cycle (Parker et al., 2003; Blasco-Costa & Poulin, 2017). In cases such as these, it is often difficult to differentiate between pathogen species and different pathogen life stages morphologically. Third, infections by a single pathogen might actually be rare in nature, instead co-infections by multiple pathogen strains or species are likely to be the norm (Petney & Andrews, 1998). In this context, exposure history and the timing of infection might play crucial roles in terms of host fitness and pathogen virulence (Telfer et al., 2010; Ben-Ami, Rigaud & Ebert, 2011). Fourth, pathogen prevalence may vary across space and time and, hence, these patterns need to be taken into consideration in comparisons across scales (Thompson, 2009). This can be on a small scale within a host (e.g., between tissues), or across geographical space (e.g., between populations/species). For example, comparison of host and viral population structure suggests that dispersing male bats spread the rabies virus between genetically isolated female populations (Streicker et al., 2016). Fifth, hosts and their pathogens rarely interact in isolation but rather as part of a larger ecosystem, which might modulate how a pathogen interacts with its host and vice versa (Graham, 2008).

Overall, the availability of large genomic datasets has been pivotal in untangling each of the five levels of complexity. Nevertheless, relying solely on genetic data can be misleading. While new techniques help to identify new pathogens, ecological patterns, and link the genetic structure of host and pathogen populations, the resulting data are ultimately correlational and cannot establish any causal relationships without an experimental approach. For example, sticklebacks (*Gasterosteus aculeatus*) caught in a lake harbored more macroparasites than those from a river (Wegner, Reusch & Kalbe, 2003). With only this observation, one might be tempted to conclude that the sticklebacks from lakes were more susceptible to parasitism than those from rivers. However, subsequent experiments revealed that sticklebacks from lakes were less susceptible to pathogens, but probably experienced higher pathogen exposure (Scharsack & Kalbe, 2014). This illustrates the need for experimental studies to confirm causal relationships implicated by field data. However, experiments are restricted in the complexity they can represent (Plowright et al., 2008). In conclusion, the interpretation of genetic data without a deep understanding of the host-pathogen ecology, and vice versa, can be misleading.

Genetic variation is typically studied at different levels, such as across species (Fig. 2A), across populations (Fig. 2B), within populations (Fig. 2C) or through time (Fig. 2D) to disentangle the underlying genetics of host-pathogen interactions. For this purpose, two main approaches are typically employed. On the one hand, the underlying genetic architecture can be inferred using genotype-phenotype association studies. The statistical association between genomic loci and host-pathogen phenotypes is interpreted as evidence for the underlying genetics of a given phenotype (Hirschhorn et al., 2002). On the other hand, instead of determining fitness costs of pathogens in single experiments or surveys (see also Box 1), biologists have turned to signals of natural selection over evolutionary time as recorded in host genomes (Sabeti et al., 2006). While these genome scans typically cannot directly test the causal selective agent, they do provide insight into the possible biological processes that are adapting most rapidly in host genomes (Biswas & Akey, 2006). Indeed, analysis of signatures of selection in host genomes identified pathogens as the most likely drivers of the observed patterns in a number of studies. For example, in *Drosophila*, Sackton and co-authors (2007) identified that a class of immune genes that directly interact with pathogens, such as receptor genes, exhibited a high proportion of genes under positive selection compared to genome-wide observations. Similarly, across mammals, viral interacting proteins have stronger signals of adaptation than other protein-coding genes across the genome (Enard et al., 2016), and more of these genes than expected by chance are also evolving by positive selection in birds (Shultz & Sackton, 2019).

A combination of selection scans and association studies has revealed important insights into differences in infectious disease susceptibilities, the identification of specific protective genes and alleles, and their evolutionary origin in humans, the most intensely studied organism with respect to disease (Nielsen et al., 2005; Kwiatkowski, 2005; Williams et al., 2005; Karlsson, Kwiatkowski & Sabeti, 2014; Malaria Genomic Epidemiology Network et al., 2015; Enard et al., 2016; Enard & Petrov, 2018). There have been similar advances in the understanding of the underlying genetics of natural host-pathogen systems in the wild. For example, Bourgeois et al. (2017) was able to confirm and refine previously identified quantitative trait loci that confer resistance in the planktonic crustacean *Daphnia magna* to the pathogen *Pasteuria ramosa.* Furthermore, investigations of signals of selection have identified additional genomic regions consistent with the evolution of resistance that were not identified by association approaches. Such loci present further candidates moderating the host-pathogen interactions, but without a clear association with specific phenotypic traits, evolution in response to other environmental variables correlated with pathogens often cannot be excluded (Bourgeois et al., 2017).

Despite the success of genome-wide associations and selection scans to find genomic evidence of pathogen pressures on hosts, simultaneous genomic investigation of the co-evolutionary dynamics between host and pathogen within a single system remain rare (see Fig. 1). Indeed, today, few systems have the genomic resources available to truly interrogate ongoing genomic changes between pathogen and host in parallel. One such example is described in Bartha et al., 2013, who identified linked sequence variants between humans and HIV through genome-wide-association scans. This study highlighted both host and viral loci that are potentially involved in the co-evolutionary dynamics between host and pathogen. Additionally, emerging studies of experimental evolution in the field or laboratory, or multigenerational sampling of natural populations of hosts and pathogens, have successfully identified novel adaptive alleles in both hosts (Scanlan et al., 2015; Cairns et al., 2017) and pathogens (Pal et al., 2007). The reciprocal nature of the interactions between host and pathogens over time naturally lead researchers to ask whether host and pathogen species co-diversify over evolutionary time and to what extent genomics can inform the underlying processes. Indeed, attempts to detect co-speciation among hosts and pathogens date back to the beginning of the 20th century (reviewed in Vienne et al., 2013). However, inferring co-evolutionary history through comparisons of host and pathogen phylogenies is challenging. For example, such comparisons can mistake a host shift followed by co-diversification as co-speciation (Vienne, Giraud & Shykoff, 2007). The former mechanism is more consistent with empirical data that suggests that the level of co-evolution necessary to drive co-speciation of host and pathogen is rarely encountered in nature (Vienne et al., 2013). As models of molecular adaptation and gene tree evolution improve, we may be able to identify phylogenetic congruence at the gene-tree level or signatures of selection that co-vary among hosts and parasites with more confidence. In turn, we might be better able to interpret results in the light of co-diversification versus co-speciation of studies, such as by Tso et al. (2018), where a pathogenic strain of *Candida albicans* evolved into a gut symbiont in mice in just ten weeks. Parallel genomic analyses of the pathogen showed that genes involved with an important virulence factor in *C. albicans*, the hyphal morphogenesis program, had undergone rapid degeneration via both point mutations and deletions.

### From gene fragments to whole genome analysis

As outlined above, the co-evolution of host and pathogens can result in distinct and measurable genomic signatures of selection, which can reveal the genetic mechanisms by which hosts and their pathogens interact. The genomics revolution has spurred the transition from single-candidate gene studies to genome wide analyses of hosts and pathogens. Historically, a number of different candidate host immune genes families have attracted particular attention for studies of host-pathogen interactions, including components of the innate immune system such as toll-like receptors (TLRs) (Tschirren et al., 2013; Zhang et al., 2014; Zhang, Lun & Tsui, 2015; Shan et al., 2018), interferons and antimicrobial peptides (Clark & Wang, 1997; Tennessen, 2005; Franzenburg et al., 2013; Carlin et al., 2018). These and other studies show, both in vertebrates and insects, widespread signatures of positive selection and rapid evolution in genes of the innate immune system (Świderská et al., 2018; Harpur et al., 2019; Adrian et al., 2019). Gene expression studies have also revealed widespread activation of host innate immune genes upon natural or experimental infection with pathogens, such as *Pseudomonas* and *Daphnia* (Kumar et al., 2018). As such, these studies have contributed much to our general understanding of the host’s responses to pathogen exposure and common pathways to resistance evolution over time.

The candidate gene family that has attracted the most attention in ecological and evolutionary host-pathogen studies, at least in vertebrates, is the major histocompatibility complex (MHC) (Hughes & Nei, 1988; Bernatchez & Landry, 2003; Meyer-Lucht & Sommer, 2005; Spurgin & Richardson, 2010). MHC genes encode cell–surface molecules that play a central role in pathogen recognition as part of the adaptive immune response. T-cells act to destroy infected cells both directly, as cytotoxic T-cells, and indirectly, as T-helper cells which activate other immune cells, but they can only determine what is self or foreign from peptides presented by MHC molecules. The number of MHC gene copies carried by individuals varies widely between, and even within, species (Kelley, Walter & Trowsdale, 2004; Cheng et al., 2012; Lighten et al., 2014; reviewed in O’Connor et al., 2019). Additionally, the allelic diversity recorded within and between gene copies makes the MHC genes the most polymorphic loci to date (Reche & Reinherz, 2003; Robinson et al., 2015). This exceptional polymorphism is believed to be primarily maintained by selection from a wide range of pathogens (Prugnolle et al., 2005; Qutob et al., 2011). Overall, it is clear that MHC genes play a pivotal role in the fight against pathogens and numerous studies have established associations between MHC genotypes and infections with a particular pathogen (Kaslow et al., 1996; Meyer-Lucht & Sommer, 2005; Oliver, Telfer & Piertney, 2009; Bolnick & Stutz, 2017; O’Connor et al., 2019).

Although the candidate gene approach has been the standard method for studying immune genes in the context of host-pathogen interactions, the rapidly decreasing costs of high throughput sequencing are making whole-genome approaches much more feasible. Whole-genome population genetic and comparative genomic studies allow unbiased detection of regions of the genome that are evolving non-neutrally across a variety of time scales. When combined with functional annotations or association studies, such comprehensive genome-wide surveys permit incisive tests of the effects of pathogens on host genomes that are simply not possible from candidate gene studies. Furthermore, whole genome studies are not restricted by *a priori* predictions of which genes are important in responding to pathogen challenges. Thus, whole-genome approaches offer the potential to reveal new unbiased insights into the genetic basis of host-pathogen interactions, e.g., (Enard et al., 2016; Shultz & Sackton, 2019). Since multiple genes are most often involved in a host’s response to a particular pathogen, whole-genome approaches also have the potential to reveal these understudied polygenic responses (Daub et al., 2013). However, a major caveat associated with the whole-genome approach is that genomic regions of high repeat content, or highly duplicated genes, often do not assemble well or at all, whether considering host or pathogen. This is particularly problematic in the case of *de novo* genome assemblies in non-model species (Peona, Weissensteiner & Suh, 2018). Genome assembly problems may be a significant disadvantage for host-pathogen studies given that some key genes which play a role in innate and adaptive immunity are not only highly duplicated but also to some degree physically linked in the genome, such as the beta-defensin and MHC genes (Kaufman et al., 1999; Hellgren & Ekblom, 2010; Balakrishnan et al., 2010). Improved sequence and scaffolding techniques are being developed to remedy problems of assembling such regions (Dilthey et al., 2015) and some may be possible to overcome with long-read sequencing methods (Peona, Weissensteiner & Suh, 2018). Thus, while the whole-genome approach may become a gold standard for many host-pathogen studies in the future, we currently see a continued need for sequencing methods that target focal genes or a reduced representation of the whole genome, in particular in studies of non-model species.

### Genomic detection and surveys of pathogens

Ever since the invention of the polymerase chain reaction, molecular approaches have continuously provided sensitive methods for the detection of pathogens, often without prior separation from the host tissues (e.g., malaria pathogens Snounou et al., 1993; Hellgren, Waldenström & Bensch, 2004). High throughput sequencing techniques have now become pivotal for both detection and identification of new pathogens, especially in cases of emerging infectious diseases, and in pathogens with complex life histories and co-infections (Blasco-Costa & Poulin, 2017). Furthermore, unmapped reads in host genome projects are likely a fruitful source of undiscovered pathogens (Laine et al., 2019). Improved pathogen genomics holds great potential to advance our current understanding of host-pathogen interactions in several ways: from an epidemiological perspective, it allows one to reconstruct the spatial spread of pathogen invasion, illuminates pathogen population dynamics, and enables forecasting of future infection scenarios. Although this has been possible previously by using only a few key genetic markers from samples that spanned decades in time, such as in studies of influenza or rabies virus (Biek et al., 2007; Streicker et al., 2016), whole genome information now allows for high-resolution characterization of outbreaks over shorter timescales (e.g., Ebola (Dudas et al., 2017) and Zika (Faria et al., 2017; Grubaugh et al., 2017)). In addition, open source genomic data-sharing sites and analysis platforms like nextstrain (http://www.nextstrain.org/) are invaluable to explore pathogen time-space variation in real-time. Moreover, genomic analyses of dated pathogen samples have proven successful in inferring directionality of pathogen spread, for example, among wildlife and livestock, thus informing effective control measures (Kamath et al., 2016).

However, many technical challenges still remain for such approaches, especially in situations where pathogens cannot be physically separated from hosts (see Box 1 for an example). For example, pathogen DNA typically makes up only a small fraction of the total extracted DNA from samples of infected hosts, and host samples must therefore be sequenced at an immense depth to obtain even a low coverage of the pathogen (Videvall, 2019). A large number of enrichment protocols for high-throughput sequencing methods have been developed to facilitate the detection and quantification of pathogens. These enrichment protocols are often efficient ways of increasing the ratio of pathogen to host DNA. Before DNA extraction, intracellular pathogens can sometimes be isolated from infected host cells using cell-sorting or laser-capture microscopy techniques (Saliba et al., 2014; Wang et al., 2015), or separated from the host cells by targeting different life stages (e.g., gametes, spores) (Palinauskas et al., 2013). Also, the process of DNA extraction itself can be tailored to significantly enrich pathogen DNA by selective lysis of host cells (Bachmann et al., 2018). This has been successfully demonstrated in the human clinical setting: saponin treatment selectively lyses human cells and thus enriches DNA concentration in mixed samples of diverse communities of microorganisms with an intact cell wall (Hasan et al., 2016). After DNA extraction, selective whole-genome amplification can specifically enrich for pathogen sequences in various ways: (i) by using oligos that are more abundant in the pathogen genome (Melnikov et al., 2011); (ii) by targeting differences in methylation between host and pathogen genomes (Gómez-Díaz et al., 2012); (iii) by sequence capture enrichment protocols for pathogen DNA (Tagle et al., 1993); or (iv) by Nanopore Cas9-targeted sequencing, a selective ligation of sequence adaptors at cut sites of Cas9 (Gilpatrick et al., 2019). Lastly, real-time evaluation of sequence data such as is possible with Nanopore technology could be further exploited to enrich for pathogen sequences during active sequencing (Edwards et al., 2019). When enrichment protocols are not feasible, host and pathogen associated reads can often be separated in silico using reference sequence databases. In such cases, low-coverage detection of genome fragments of pathogens in host genome sequencing reads is a straightforward and fruitful approach (e.g., Laine et al., 2019). Using this approach, putative RNA viruses of *Drosophila melanogaster* were identified from de novo assembled RNAseq reads (Webster et al., 2015). Dual sequencing analysis of both host and pathogen can be further exploited to characterize the physiological response throughout the course of an infection (Florens et al., 2002; Jean Beltran et al., 2017). However, sequencing coverage and cost are major factors determining feasibility and scope of a study. Enrichment and optimization of protocols carry the caveat that they are study specific and, in many cases, not universally applicable.

Simultaneous genome sequencing of multiple species—metagenomics—can help the field expand beyond the two-organism framework (Westermann, Barquist & Vogel, 2017), as has been most extensively demonstrated in microbiome research in the context of host health (Sekirov et al., 2010; Kamada et al., 2013). It is now clear that the whole microbial community shape host health, but are also in turn selected for and manipulated by hosts (Näpflin & Schmid-Hempel, 2016; Schwarz, Moran & Evans, 2016; Rolhion & Chassaing, 2016; Näpflin & Schmid-Hempel, 2017). In particular, metagenomics is increasingly able to shed light on the function of individual members of the microbiome, for example, by investigating metabolic pathways present in the community (Lee & Hase, 2014). Similarly, sophisticated pathogen-specific screening tools such as sequence chips with known pathogen probes can effectively screen complex ecosystems for pathogens within the community and may identify potential disease reservoirs (Bird & Mazet, 2018). Such approaches are employed by the PREDICT project of USAID which attempts to identify new zoonotic threats in “hot spot” regions in Africa, Asia, and Latin America by sampling wildlife (particularly non-human primates, bats, and rodents) as well as people with close contact with wildlife (http://www.vetmed.ucdavis.edu/ohi/predict/).

Overall, genome-wide techniques and approaches provide us with an unprecedented wealth of information upon which specific hypotheses can be formulated and experimentally tested. A lingering limitation to the impact of such studies is low quality and poor annotation of reference genomes, especially for non-model host species. This challenge considerably slows our rate of discovery because many important parts of the host genome that respond to pathogen infection may remain undiscovered if they do not assemble properly or lack known gene annotation. Furthermore, relevant links to host-pathogen interaction could be missed because the link between genetics and the expressed phenotype is only poorly understood (e.g., the layer of mucus covering the mucosal surface in vertebrates whose composition is relevant for the specific host-pathogen interaction; see Box 2).

BOX 2 Barriers to infections—an example of difficulties linking genotype and phenotype.

**Figure 3:**
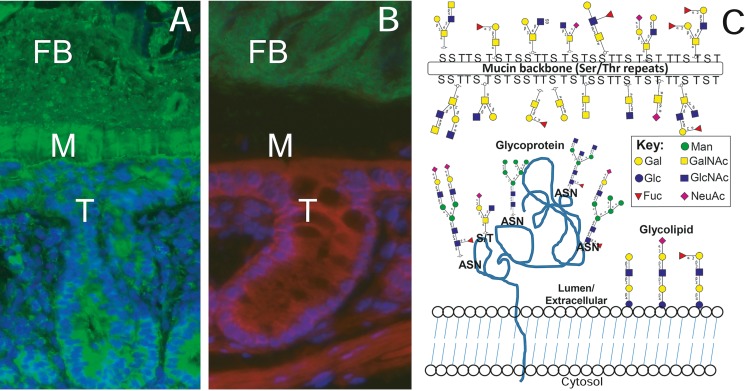
The mucosal surface. Photomicrographs (A and B) show two staining variants of the colonic mucosal tissue (T) of a healthy mouse, where a mucus layer (M) keeps the majority of the fecal bacteria (FB) from direct contact with the surface of the epithelial cells. In panel (A), the Muc2 mucin (the main component of the mucus layer) is stained in green and nuclei from the eukaryote cells in the tissue are stained blue. Muc2 is produced by cells in the mucosal tissue, secreted into the mucus layer, and present in degraded form in the fecal material. In panel (B), the mucosal epithelial tissue is outlined with red, eukaryotic nuclei are purple, the mucus layer unstained (but clearly visible due to the absence of bacteria) and the bacteria are labelled green. Panel (C) gives an overview of glycan structures that build the mucus layer and glycocalyx. Glycolipids and glycoproteins are anchored in the eukaryotic cell membrane, and secreted mucins are highly glycosylated glycoproteins consisting of 70–90% of glycans that make up the bulk of the mucus layer. The glycans can be longer and more complex than depicted in this illustration. The glycans can be either N-linked (via Nitrogen in asparagine) or O-linked (via Oxygen in serine or threonine) to the protein core, and these two types of glycan chains differ with regards to biosynthetic pathway and structure. Photo credit: Sinan Sharba.

Hosts are continuously exposed to potential pathogens, yet the establishment of an infection upon encounter is a relatively rare event. Most pathogenic infections are successfully prevented by “simple” barriers, the host’s first lines of defense (McGuckin et al., 2011; Hall, Bento & Ebert, 2017). One of the most underappreciated pre-infection barriers in (non-human) ecology is the continuously secreted layer of mucus covering the mucosal surface in vertebrates (Fig. 3A), and the glycocalyx that covers other epithelial cells and surrounds some single celled organisms (Quintana-Hayashi et al., 2018). As opposed to the skin, which is a dry, acidic, and of much smaller surface, the mucosal surface is orders of magnitude larger and presents a semipermeable, humid environment that many bacteria and pathogens could thrive in. However, the mucosal surfaces are protected by several layers of defense that a pathogen has to circumvent to either gain access to close interactions with host cells, or entry into host cells, or transferring across the host epithelium. The first barrier the pathogens encounter is the continuously secreted mucus layer covering the cells and the epithelial glycocalyx (Quintana-Hayashi et al., 2018), into which a range of antimicrobial molecules are secreted, the bulk of this layer consists of a massive amount of highly diverse glycans (Fig. 3B). Among these highly diverse glycoconjugates, there are those that act as protection against infection by binding and disseminating the pathogen, act as steric hindrance or releasable decoys, but also those that act as receptors for pathogens and confer intimate adherence (Linden et al., 2008a; Lindén et al., 2008b; Lindén et al., 2009; Padra et al., 2018).

In fact, across mammalian and teleost species, most known interactions between viral or bacterial pathogens and their hosts occur via host glycan structures (Aspholm-Hurtig, 2004; Linden et al., 2008a; Lindén et al., 2008b; Venkatakrishnan, Packer & Thaysen-Andersen, 2013; Padra et al., 2014; Skoog et al., 2017). Interactions between host glycans and pathogens are thus central for host-pathogen specificity and virulence. As such, one would expect that host glycans and pathogen adhesins are subjected to strong selective pressure (Linden et al., 2008a; Lindén et al., 2008b; Linden et al., 2010, 2010; Vitiazeva et al., 2015; Venkatakrishnan et al., 2017; Venkatakrishnan et al., 2019). While certain individual interactions between host glycans and pathogen adhesins have been dissected in detail (Rydell et al., 2011; Bugaytsova et al., 2017) it remains difficult to actually identify different glycoconjugate compositions and their underlying genetic basis.

While enzymes involved in glycan biosynthesis are easily identified based on sequence identity (curated collection: http://www.cazy.org; Lombard et al., 2013) and make up about 5% of the total genome (Rini, Varki & Esko, 2015) the resulting glycan structures are governed by stochastic events, substrate availability and state of differentiation and physiological environment. Thus, with the currently available knowledge it is not feasible to predict glycan repertoire and biosynthetic machinery based solely on genomic and/or transcriptomic sequence data of the host. In addition, we currently lack the ability to screen large sample sets for glycan repertoire because mass spectrometric based glycomics discovery is at its best only semi-automatic. Additionally, on the pathogen side, most adhesins of pathogenic organisms have yet to be identified and characterized. In conclusion, advances in the biological understanding of the system and technological innovation likely need to go hand-in-hand with functional validation of the underlying genetic basis to advance the genotype-phenotype mapping of glycan structures generally, and particularly in the host-pathogen context.

### Infrastructural challenges of generalizing results across species and systems

The genomic data revolution driven by high-throughput sequencing has created numerous exciting opportunities to study host-pathogen interactions in a multitude of systems in unprecedented detail. This revolution extends to non-model organisms, although inference here may be hampered by a lack of suitable and/or sufficient host or pathogen samples. Moreover, even when such obstacles can be overcome, two major problems of relevance to this review continue to constrain the full impact, reuse, synthesis and reproducibility of host-pathogen studies, particularly for non-model systems: both involve the deposition and analysis of associated sequence data. First, while it should be standard practice to deposit at a minimum raw sequence data in well-curated, detail-rich national databases such as the National Center for Biotechnology Information (NCBI), the European Nucleotide Archive (ENA), or the DNA Data Bank of Japan (DDBJ), the associated biological metadata of these samples are often inadequate or misleading due to various inconsistencies in available sample information that is being deposited. This problem is not trivial to resolve in the context of host-pathogen studies, in part because the complexity of the standardized metadata forms of these platforms for deposition and retrieval of data (Dugan et al., 2014; Chang et al., 2016; Singh et al., 2019). Second, the analysis of genomic data is preceded by a large number of computationally complicated pre-processing steps. The choice of algorithm and parameters in this pre-processing procedure can often have significant impacts on the final results but are generally inadequately documented and communicated. Together, the missing metadata and the lack of transparency regarding computational tools confound or even prevent robust meta-analysis and comparative studies; and without meta-analyses and comparative studies, results from individual studies of various host-pathogen systems cannot be integrated into a larger context.

Improving the availability of metadata and the transparency of computational tools requires researchers to collect and analyze their data in an open format, with the goal of making the data and the methods publicly available and useable for comparative studies. Because the interactions between hosts and their pathogens are inherently dynamic across space and time, accurate information on sampling location and timing is essential information to include in metadata. This includes the host source of isolation for pathogens and the infection prevalence of hosts. Importantly, the nomenclature of genetically identical strains or species must be consistent. For example, despite being genetically identical the haemosporidian lineage “*Haemoproteus* lineage 22” from birds, first described in 2002, has repeatedly been named differently in publications appearing between 2002-2009: “AP21”, “COLL2” “SWTH.H2”, and “WHA24” (Bensch, Hellgren & Pérez-Tris, 2009). An obvious first step is to improve the design, user-friendliness and programming interface of existing popular databases for metadata. An integration of a large amount of data sources has been developed for some systems, such as haemosporidian pathogens in birds (MalAvi database, Bensch, Hellgren & Pérez-Tris, 2009), or influenza viruses (GISAID database, Yuelong Shu, 2017). Such efforts should ideally be extended to all host-pathogen systems and are being realized more systematically under the umbrella of The Eukaryotic Pathogen Genomics Resource (EuPathDB), a database of pathogen genomic data that currently includes a dozen pathogen groups (Aurrecoechea et al., 2017).

Similar to metadata documentation, detailed documentation of sample processing and ultimately the choice of data analysis tools and parameter settings are becoming more widely advocated (Nature Editorial, 2017). Hence, appropriate workflow documentation is essential and will become an important component of comparative biology in the genomic era in general and in particular in host-pathogen interaction studies. This begins with explicit wet lab protocol documentation that can be easily referenced in publications, such as protocols.io (Teytelman et al., 2016), a protocol repository. This complements other peer-reviewed options from journals specifically dedicated to methods publication, such as *Nature Protocols*, *JOVE*, or *MethodsX*. For data analysis, the use of scripted pipelines and version-controlled analyses has been advocated to address challenges of analysis reproducibility (e.g., Nunez-Iglesias, 2015). At the most basic level this includes a scripted analysis that does not require manual command input and thus is completely repeatable given the same raw data and sufficient computational time (Beaulieu-Jones & Greene, 2016). Today, various toolkits, repositories and work platforms exist that advocate these principles and facilitate their implementation (see https://github.com/pditommaso/awesome-pipeline for a non-exhaustive but curated list). Among others, these include literate programming options such as provided by R Markdown (https://rmarkdown.rstudio.com) or Jupyter (https://jupyter.org), code development repository such as GitHub (https://github.com) or Dryad (https://datadryad.org), as an example of a more general digital repository. For genomics specifically, the graphical user interface guided data integration, analysis, and publishing platform Galaxy has been a long-time advocate of communicating standardized best practices of analysis workflows and thus ensuring reproducibility and development of common analyses pipelines. Overall the adoption of best practices and detailed workflow documentation will improve reproducibility and integration of results across studies, however, it does not preclude the careful selection and validation of appropriate methods (Lotterhos, Moore & Stapleton, 2018). In principle, this could ultimately lead to automated analysis of organisms with more limited genomic resources, which might permit linking of metadata (such as whether a study is experimental or naturally observed) with sequence data across studies. Such examples are currently still mainly restricted to curated data sets with a narrow purpose, for example Bgee (Bastian et al., 2008) which facilitates automated cross-species comparison of “healthy” control individuals. Extensions of such projects would open up exciting frontiers in comparative studies of host-pathogen interactions across different systems and beyond. At this time, however, comparative studies such as the recent investigation into MHC copy number variation across *Aves* (Minias et al., 2018) illustrate the norm: researchers evaluate large amounts of data from repositories, which they curate by consulting the primary publication for a specific question, and statistically account for inconsistencies and uncertainties of the assembled data in their analysis.

### Studying host-pathogen interactions in the Anthropocene

The number of pathogen infections is predicted to continue to increase in the near future, as climate change, human population growth and transportation impact the geographic distribution and contact rate of hosts and pathogens (Altizer, Bartel & Han, 2011; Harding et al., 2012; Maganga et al., 2014; Snäll et al., 2015). This applies to any type of host: human, animal, plant, etc. It has been estimated that wildlife is the source for 72% of emerging infectious diseases in humans (Cleaveland, Laurenson & Taylor, 2001; Jones et al., 2008; Olival et al., 2017) with recent examples including SARS, a virus in bats and small mammals; the avian influenza type H5N1; and Ebola, originally a virus in fruit bats, which recently caused a human catastrophe in western Africa (Dudas et al., 2017). Obviously, such pathogens can have wide-ranging consequences on global societal stability and economy, and can have devastating effects on natural populations (Daszak, Cunningham & Hyatt, 2000; Harding, Härkönen & Caswell, 2002; Sachs & Malaney, 2002; Bonds et al., 2009). In this context, rapid DNA sequencing technologies offer great promise for our understanding of host-pathogen dynamics, and hence the ability to predict and control disease epidemics (Wohl, Schaffner & Sabeti, 2016; Takahashi et al., 2018).

Natural systems are increasingly subjected to anthropogenic stressors, including climate change, urban development, overexploitation, pollution, noise, and transport (Gerber et al., 2014). In recognizing that no host-pathogen system exists in isolation, it is essential to understand how such stressors affect the host’s fitness, immune system and pathogen susceptibility. For instance, immunotoxic contaminants can have substantial population level effects by contributing to anthropogenic stress and infectious disease outbreaks (Desforges et al., 2016). This is particularly true for marine and terrestrial top-predators, which, due to their life-history and placement at the top of the food chain, accumulate high levels of ecotoxins. Indeed, high tissue concentrations of persistent pollutants in Baltic seals in the 1970–80s were associated with oviduct occlusions and impaired immune system, leading to sterility and repeated infections (Bergman & Olsson, 1986), and recent work suggest that the same may be true for a wide range of European dolphins and killer whales (Jepson et al., 2016). Such increased levels of ecotoxins may explain the increasing prevalence and severity of diseases in marine wildlife (Härkönen et al., 2006). A detailed understanding of the role of these and other stressors in host-pathogen systems will require multispecies and multi-methodological approaches integrating information at all levels of the system, including trophic interactions, resource availability, life-history and population dynamics, as well as gene expression and selection.

Human intervention also has the potential to alter pathogen communities directly, both by eliminating and by introducing pathogens (Daszak, Cunningham & Hyatt, 2000). Pathogens can play crucial roles as ecosystem engineers (Thomas et al., 1999; Wood & Johnson, 2015). Often, we lack the knowledge to accurately predict how the elimination of one pathogen will affect the host population, other pathogens within the same host population, and their effect on the ecological community (Rogalski et al., 2017). For example, the introduction of invasive species often inadvertently results in the introduction of novel pathogens against which native hosts may possess little or no protection (Van Riper et al., 1986). Here again, major future challenges include sample availability, ecological monitoring, and the collection and deposition of appropriate metadata. Additionally, cross-disciplinary scientific integration and communication between scientists, managers and decision-makers are crucial in order to advance global health.

## Conclusions and Prospects

Innovations in genomic techniques have the potential to bring a synthesis to the study of host-pathogen interactions across systems and environmental conditions. We highlighted several recent trends in this perspective for genomic studies of host-pathogen systems: (i) evolutionary genomics approaches have allowed the field to move from a candidate gene approach to investigations at the scale of whole genomes; (ii) the use of genomics for the detection and surveillance of host-pathogen systems; (iii) the challenges of the integrating natural history and ecological metadata and genomic data across systems and timescales due to infrastructural challenges of database integration and transparency; and (iv) the impact of anthropogenic stressors on host-pathogen systems that have consequences for global health. Additionally, our survey of the recent literature of ecological genomics of host-pathogen interactions revealed that studies with spatially and ecologically complex settings are rare, as are detailed studies of host genomic responses to pathogens. Any single host-pathogen study is constrained by limited resources or genomic tractability, the geographical and evolutionary time scales involved as well as environmental complexities. Accordingly, transparent and open science will help to achieve a comprehensive understanding of host-pathogen interactions in general. This will contribute to the integration of findings across the different scales (Fig. 1). A large repertoire of comparable and inter-communicative studies will facilitate a more generalizable understanding of the causes and consequences of host-pathogen interactions and a clearer roadmap to combating the continuous threat of pathogens in a changing world.

##  Supplemental Information

10.7717/peerj.8013/supp-1 Table S1 References and scoring results of literature survey Literature search on Web of Science (accessed August 30, 2018) with the following search query: (host-parasite* OR host-pathogen*) AND (genomic*). We refined the search hits by document type to include only articles, covering the publication years 2014–2018, and excluding studies with no genomic aspect. The table contains 263 papers with scores for each scale defined in Table 2.

## Additional Information and Declarations

### Data Availability

The following information was supplied regarding data availability: The scoring data and references for the literature survey is available in Table S1.
